# Myxoid glioneuronal tumour – report of three cases of a new tumour in a typical location and review of literature

**DOI:** 10.1259/bjrcr.20200139

**Published:** 2021-02-04

**Authors:** Eduardo de Oliveira Narvaez, Bruno Shigueo Yonekuro Inada, Paulo Ricardo Sousa Frota de Almeida, Leonardo Furtado Freitas, Matheus Dorigatti Soldatelli, Danilo Manuel Cerqueira Costa, Victor Hugo Rocha Marussi, Christiane Siqueira Campos, João Luiz Vitorino Araujo, Henrique Carrete Junior, Lázaro Luis Faria do Amaral

**Affiliations:** 1 Department of Neuroradiology, BP Medicina Diagnóstica, Hospital Beneficência Portuguesa de São Paulo, São Paulo, Brazil; 2 Department of Neuroradiology, Universidade Federal de São Paulo (UNIFESP), São Paulo, Brazil; 3 Discipline of Neurosurgery, Faculdade de Ciências da Santa Casa de São Paulo, São Paulo, Brazil

## Abstract

Formerly called dysembryoplastic neuroepithelial tumour (DNET) of the septum pellucidum, myxoid glioneuronal tumour (MGT) was recently recognized as a distinct entity. We report three cases of presumed MGT with typical location and image features.

## Introduction

In 2020, Louis et al by the cIMPACT-NOW update 6, the Consortium to Inform Molecular and Practical Approaches to Central Nervous System (CNS) Tumour Taxonomy, recommended “new” entities and revised definitions of “old” entities, both paediatric and adult, on future CNS classification and grading.^1^ In this meeting, a new entity called Myxoid Glioneuronal tumour (MGT) was proposed based on the anatomic location, imaging features and genetic mutation.

It was defined that MGT is a circumscribed, slow-growing mixed neuronal-glial that characteristically involves the septum pellucidum, septal nuclei, subcallosal area, corpus callosum and lateral ventricles, and was formerly referred to as dysembryoplastic neuroepithelial tumour (DNET) of the septum pellucidum in the past, because of the histological features that resemble the typical cortical DNET.^1–4^

In this case series, we describe three cases of MGT, two of them with histological confirmation. Besides, we present a literature review of the clinical profile, image findings and histological features of this new central nervous system tumour.

## Cases reports

### Patient 1

A 59-year-old female came to the emergency department with complaints of chronic headaches. The neurological examination was unremarkable. The patient was referred to the radiology department for a non-contrast CT scan, which showed a hypodense, well-delimited lesion in the right septal area with no calcification or haemorrhage.

**Figure 1.**
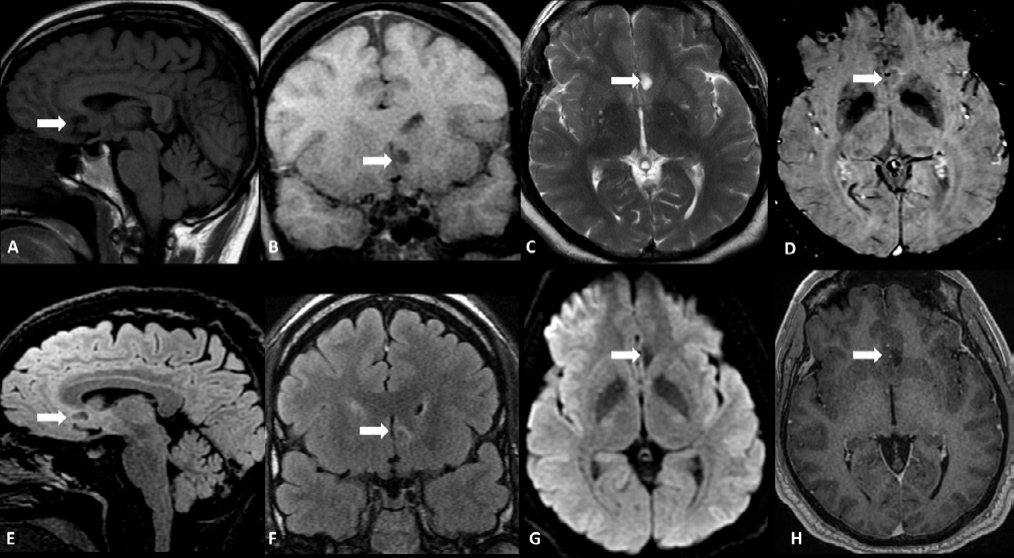
A 59-year-old female with chronic headache. Conventional MRI shows a round-shaped infiltrative lesion in the left septal/subcallosal area (white arrow) with a hypointense signal on Sagittal (**A**) and Coronal (**B**) T1W and hyperintense signal on T2WI (**C**). Note the central hypointense and peripheral hyperintense signals on both SWI (**D**), Sagittal (**E**) and Coronal (**F**) FLAIR. The lesion demonstrates facilitated diffusion on DWI (**G**), and no contrast-enhancement on Axial CE-T1WI is seen (**H**).

**Figure 2.**
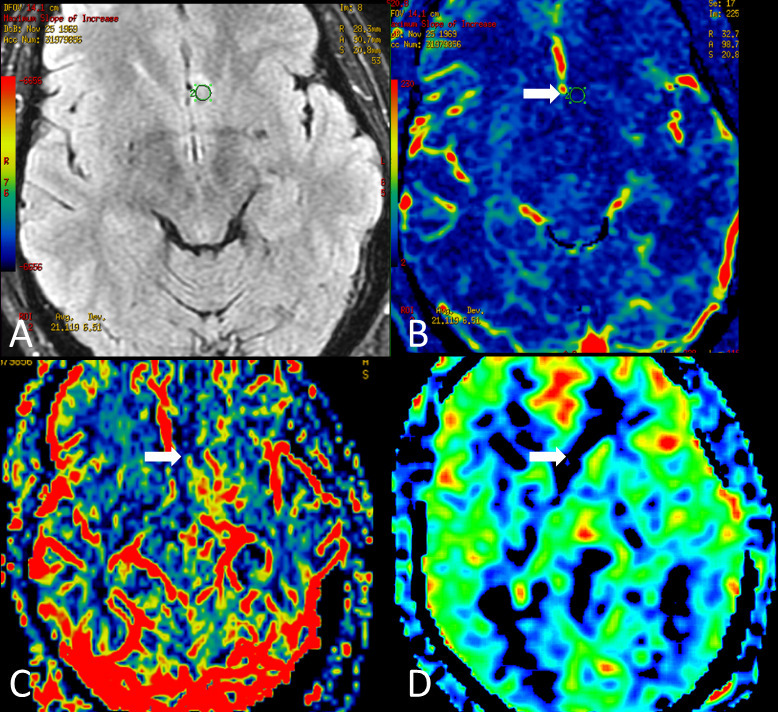
Perfusion MRI of the same patient. Axial FLAIR (**A**) localizes the tumour. Dynamic Contrast-Enhancement (DCE) permeability map (**B**) shows a standard map of Maximum Slope of Increase. Dynamic susceptibility contrast (DSC) rCBV and Arterial Spin Labeling CBF maps (C, and D, respectively) demonstrate normal capillary density and cerebral blood flow, compatible with a hypoperfused lesion.

Brain MRI revealed a well-defined nodular lesion in the left subcallosal gyrus (septal area) with hypointensity on *T_1_* weighted image (T1WI), hyperintensity on *T_2_* weighted image (T2WI) and no gadolinium enhancement. On fluid attenuation inversion recovery (FLAIR) image, the lesion showed central hypointensity with a periphery rim of hyperintensity. Diffusion-weighted image (DWI) showed a hypointense signal with a corresponding high signal on the ADC map (facilitated diffusion) and a slight hypointense signal in the susceptibility-weighted image (SWI) (Figures 1 and 2). Arterial spin-labeling (ASL), dynamic susceptibility contrast (DSC) and dynamic contrast-enhanced (DCE) MR perfusion confirmed a hypoperfused lesion (Figure 2).

The patient is currently under conservative management in neurology clinics, and no surgical intervention was performed.

### Patient 2

A 49-year-old male was referred for a 3-month headaches history and nausea, associated with behavioural abnormalities, and two episodes of simple partial seizures. Brain MRI showed a well-defined lobulated lesion in the subcortical white matter of the right subcallosal gyrus and lamina terminalis, with the chiasmatic and infundibular involvement recesses and extension to the nucleus accumbens and anterior commissure.

**Figure 3.**
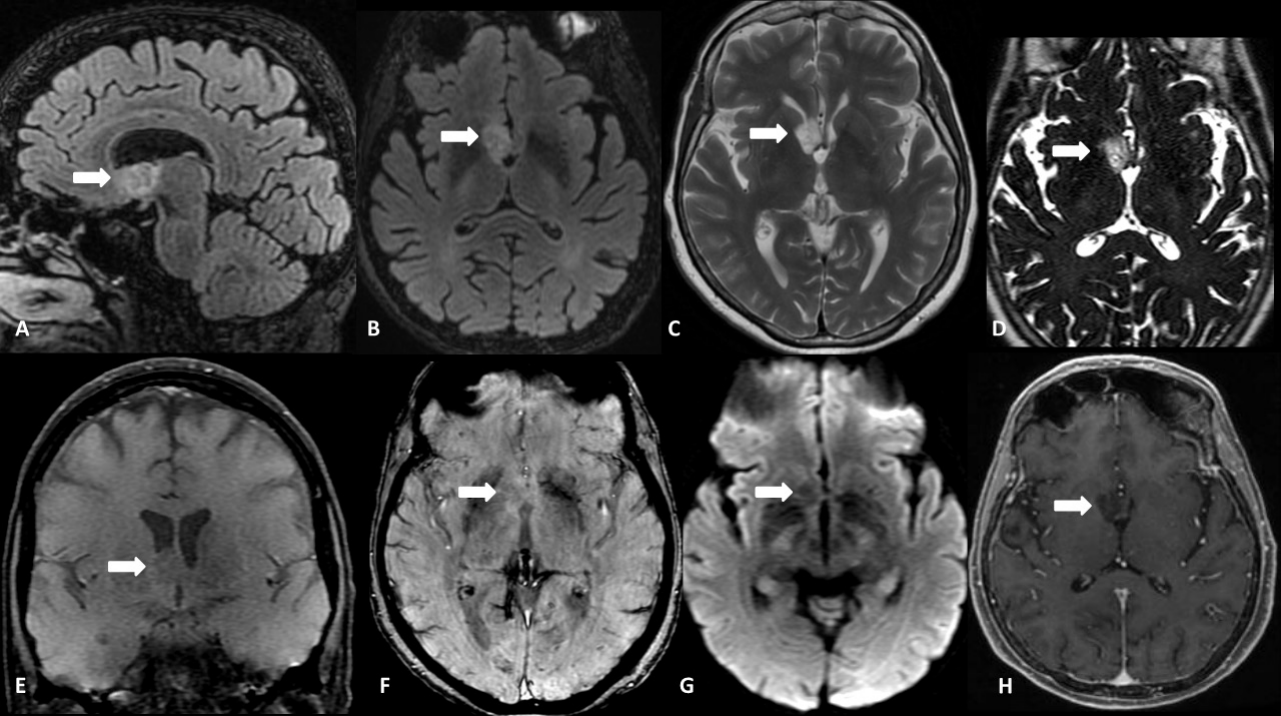
Male, 49 years old, presenting with progressive episodes of visual abnormalities and loss of consciousness. Conventional MRI show an infiltrative lesion (arrows) in the right subcallosal gyrus (septal area) and lamina terminalis, with the involvement of the chiasmatic and infundibular recesses, ipsilateral nucleus accumbens, and anterior commissure. Sagittal (**A**) and Axial (**B**) FLAIR demonstrate a bright rim with a partially suppressed central area, and Axial T2WI (**C**) shows a prominent hyperintense signal. Axial 3D-CISS (**D**) depicts a high inhomogeneous signal, confirming that the lesion is not cystic. There is a hypointense signal on both T1WI (**E**) and SWI (**F**) and facilitated diffusion on DWI (**G**). No abnormal enhancement is seen on Axial CE-T1WI (**H**).

**Figure 4.**
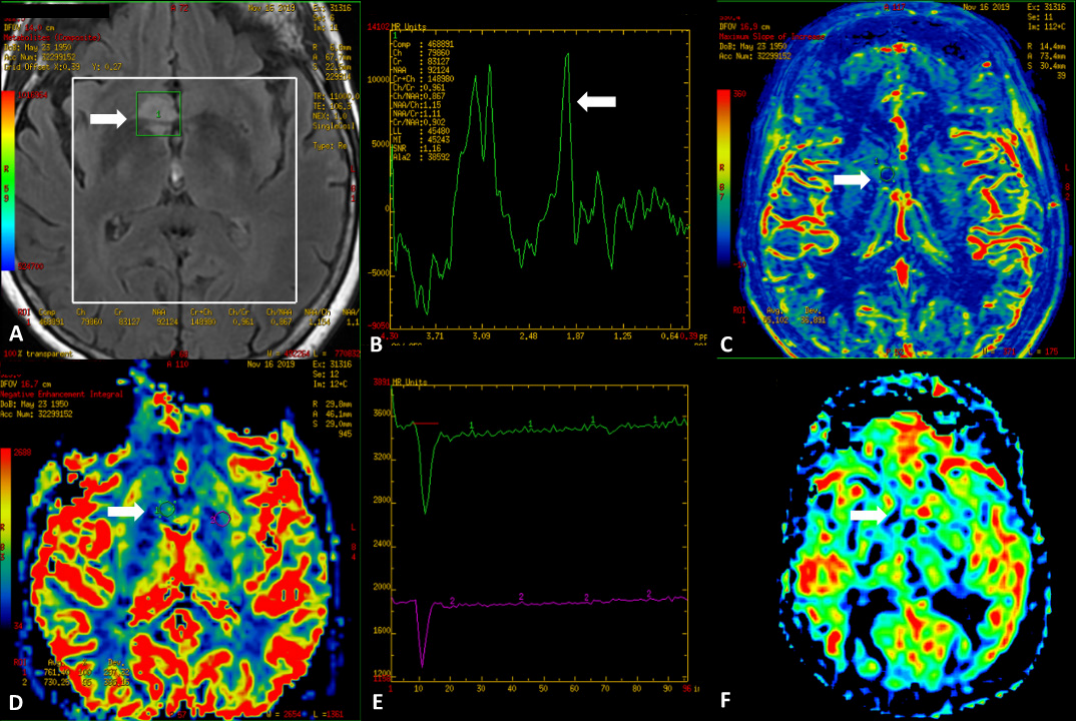
Advanced MRI of the same patient. Axial FLAIR (**A**) localizes the tumour. MR spectroscopy (**B**) shows a mild decrease in the NAA peak (arrow). MR perfusion studies demonstrate a normal permeability map on Dynamic Contrast-Enhancement (DCE) (**C**). Low rCBV on dynamic susceptibility contrast (DSC) (**D and E**), and normal rCBF on the Arterial Spin Labeling map (**F**), indicates a low perfusion lesion.

This lesion showed hyperintensity on T2WI, a slight hypointensity on SWI and no gadolinium enhancement. There was a central partially suppressed signal with a periphery rim of hyperintensity on the FLAIR image. There were no signs of restricted diffusion, calcification or haemorrhage (Figure 3). ASL, DSC, and DCE MR perfusion confirmed a hypoperfused lesion. MR spectroscopy characterized a mild decrease in the NAA peak but no elevation in the Choline peak (Figure 4).

Considering the clinical findings, this patient underwent a microsurgery resection without complications, and the diagnosis was septal DNET. The patient was discharged home the day after surgery. At a follow-up visit 3 months after surgery, the headaches had lessened in frequency. No more seizures were related.

## Case report 3

A 22-year-old male who underwent brain MRI was reporting a 7-month history of disconnected speech and upper limb movements, along with sporadic episodes of loss of consciousness lasting approximately 15 to 20 s. These episodes increased gradually as frequently as twice a day.

**Figure 5.**
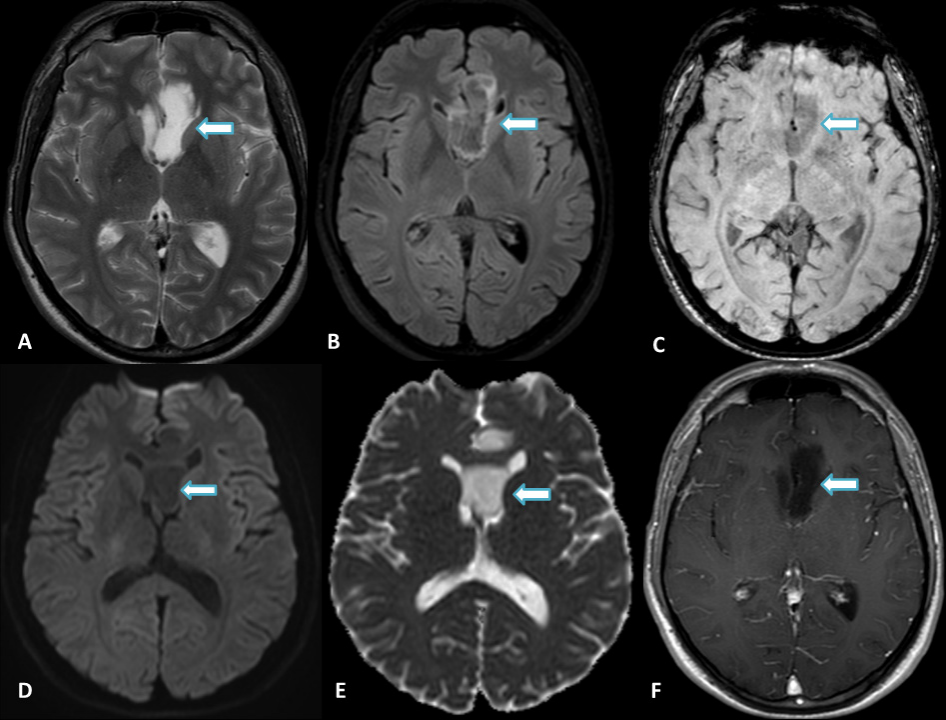
Male, 22 years old, presenting with progressive episodes of loss of consciousness, disconnected speech, and upper limb disorders started 7 months ago. Axial conventional MRI images demonstrate a large infiltrative lesion (arrows) in the left septal area (subcallosal gyrus) with a prominent hyperintense signal on T2WI (**A**), partially suppressed on FLAIR (**B**). Note also the well-defined hyperintense rim on FLAIR (**B**). SWI demonstrates low signal inside the lesion (**C**) and facilitated diffusion on DWI and ADC map, consistent with low cellularity (**D and E**). No contrast enhancement is seen (**F**).

**Figure 6.**
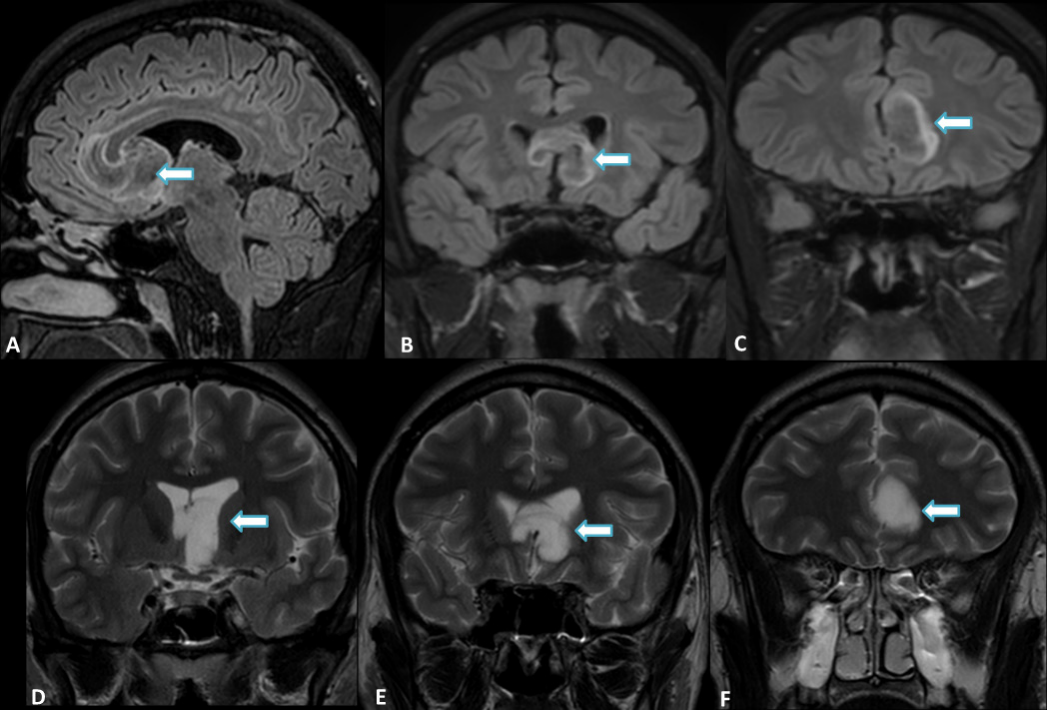
Sagittal and coronal FLAIR (**A, B, C**) and T2WI (**D, E, F**) of the same patient better characterize the large infiltrative lesion (arrows) in the left septal area (subcallosal gyrus) involving the genu of the corpus callosum, with anteroinferior extension to the septum pellucidum, left frontal horn of the lateral ventricle and ipsilateral III ventricle. The partial suppression and hyperintense rim are also shown on FLAIR. Note the “L” shaped appearance on Coronal images.

Brain MRI showed a sizeable well-delimited lesion with an “L” shape in the left septal area, inferior aspect of the septum pellucidum, with extension into the third ventricle (Figure 5). This lesion depicted a hyperintense signal on T2WI, partially suppressed with a hyperintense thin rim surrounding the tumour in FLAIR, and a slight hypointensity on SWI (Figure 6). There were no signs of restricted diffusion, calcification or haemorrhage.

The patient underwent open craniotomy with partial resection. The pathological diagnosis was septal DNET. No follow-up scan was performed until this publication.

## Discussion

The MGT was first described as a single entity in 2018 by Solomon et al that demonstrated an isolated mutation in the PDGFRA gene resulting in dinucleotide substitution at codon p.K38.^2^ Besides the PDGFRA mutation, no additional pathogenic mutations were identified.^1,2^ The cIMPACT6-Utrecht meeting suggested that MGT should be considered a distinct tumour type and could be placed within the section covering “Neuronal and mixed neuronal-glial tumours” as WHO Grade I lesion.

These tumours are characterized by the presence of neoplastic ganglion cells and glial (astrocytic or oligodendroglial) elements and represent up to 2% of all CNS tumours.^5^ The majority can be cured by surgical excision, and a minority needs radiation or chemotherapy. With few exceptions at histology, these tumours are classified as Grade I, with morphologically variable and usually occurs in children and young adults. Headache is the most common symptom, followed by seizures, emesis, behaviour disorders, visual disturbances, altered mental status, hydrocephalus, facial drop and personality changes.^1,2,6^ Some patients can be asymptomatic, and the tumour is incidentally discovered in brain imaging studies.

All three cases had a presumptive diagnosis of MGT based on the typical location and MRI features. As demonstrated by Salomon et al and suggested by cIMPACT6-Utrecht meeting, the MGT characteristically involves the septum pellucidum, septal area, subcallosal area, corpus callosum and lateral ventricle. CT does not play a significant role in TGM characterization. It frequently shows a hypodense lesion with a pseudocyst appearance, but no true cysts are seen histologically. There was no calcification or acute haemorrhage.

The conventional MRI clarifies the findings and usually shows a well-defined lobulated mass, with hypointensity on T1WI, hyperintensity on T2WI, and no gadolinium enhancement. FLAIR imaging is essential to this new entity’s diagnosis, as it shows a partially suppressed signal in the lesion center, with a peripheral rim of hyperintensity, classically described in the DNET.^7,8^ The 3D highly T2WI sequence (CISS FIESTA, BALANCE) with high liquid-brain contrast demonstrates a slightly decreased signal compared with the cerebrospinal fluid, confirming that it is a solid lesion. Diffusion-weighted image shows facilitated diffusion and helps differentiate it from the multinodular vacuolating neuronal tumour (MVNT), that was recently described as a true neoplastic process.^9,10^ TGM shows high ADC values compared with normal brain parenchyma because of their low cellularity. Lack of oedema is characteristic. Large septal lesions may show a distinct “L”-shaped appearance and can do a critical mass effect, suggesting another high-grade neuroepitelial tumour; his specific location is an excellent guide for the diagnostic hypothesis.^11^ However, often due to the compressive effect and psychological symptoms, surgery is necessary.

Another recent MRI method used to evaluate blood flow using electromagnetically labelled arterial blood water is arterial spin labeling. This technique uses radiofrequency pulses to magnetize the inflowing blood water protons.^12^ They are labelled by inverting their spin polarity (preparation phase) and a delayed time when perfusion-weighted images are acquired contrast use. ASL in the Neuronal and mixed neuronal-glial tumours do not demonstrate high CBF like in high-grade gliomas. These values are an essential non-invasive and fast parameter that helps in grading and differentiating the central nervous system tumours.^12^

The most important differential diagnosis of MGT located in the septum pellucidum is the third ventricle’s colloid cyst. The colloid cyst usually presents a hyperattenuating mass on CT, high signal intensity on T1WI, and variable signal intensity on T2WI in MRI.^13,14^ The cysts are typically attached to the anterosuperior portion of the third ventricular roof. They rarely are found at other sites, including the lateral ventricles, cerebellar parenchyma, pituitary and various extra-axial locations.^14,15^

Central neurocytoma and subependymoma are tumours that can also occur near the septum pellucidum. Central neurocytoma characteristically demonstrates a solid-cystic appearance, well-circumscribed mass**,** attached to lateral ventricles' septum pellucidum walls. Calcification is common (50%), as likely as variable and heterogeneous post-gadolinium enhancement.^6,16^ Subependymoma is a rare slow-growing tumour fourth ventricle in middle-aged to elderly patients, and when it protrudes into the lateral ventricles, more often than into the third ventricle. It usually does not enhance on post-contrast sequences; however, it is a solid glial lesion with a central hyperintense signal on the FLAIR image.^17^ Beyond these lesions, it is essential for a radiologist to know the normal anatomy and exclude a pitfall by CSF flow artefact simulating lesion.^15^

Histologically, MGT demonstrates a low-grade proliferation of oligodendrocyte-like tumour cells embedded in a prominent myxoid/mucin-rich stroma. Additionally, a fine capillary network reminiscent of DNET or oligodendroglioma is present. In some cases, it may demonstrate neurocytic rosettes.^1,2,7^ Recent studies found that immunohistochemistry neuronal components are negative to FGFR1 mutations or rearrangements, which characterize most of the cortical DNTs or rosette-forming glioneuronal tumours negativity to BRAF, RAF1 or FGFR1 mutations or fusions present in most pilocytic astrocytomas or other low-grade neuroepithelial tumour entities.^1,2,5^ The multi-nodularity typical pattern of cortically based DNET is also absent.

Despite being classified as a WHO Grade I tumour with relatively benign behaviour, intraventricular dissemination and local recurrence have been described.^1,2^ This behaviour is essential to make the correct diagnosis of MGT and its differentiation from other benign lesions, like colloid cysts, subependymomas or MVNTs.

## Learning points

1. Myxoid glioneuronal tumour is a new entity with a distinct isolated genetic mutation recently described. For that, in the cIMPACT6-Utrecht meeting, this lesion was considered a distinct tumour type. It could be placed within the section covering “Neuronal and mixed neuronal-glial tumours” as WHO Grade I lesion.
2. The interpreting radiologist should recognize the MGT’s imaging features, mainly of this specific location – septal area, corpus callosum and lateral ventricles – and this relatively benign behaviour evolution.
3. MGT’s imaging features should help differentiate him from other lesions in this area, like a colloid cyst, central neurocytoma or subependimoma.
